# A guide to enteral nutrition in intensive care units: 10 expert tips for the daily practice

**DOI:** 10.1186/s13054-021-03847-4

**Published:** 2021-12-14

**Authors:** Jean-Charles Preiser, Yaseen M. Arabi, Mette M. Berger, Michael Casaer, Stephen McClave, Juan C. Montejo-González, Sandra Peake, Annika Reintam Blaser, Greet Van den Berghe, Arthur van Zanten, Jan Wernerman, Paul Wischmeyer

**Affiliations:** 1 Erasme University Hospital, Université Libre de Bruxelles, 808 Route de Lennik, 1070 Brussels, Belgium; 2 Intensive Care Department, College of Medicine, King Saud Bin Abdulaziz University for Health Sciences and King Abdullah International Medical Research Center, Ministry of National Guard Health Affairs, Riyadh, Saudi Arabia; 3 Adult Intensive Care, Lausanne University Hospital, CHUV, 1011 Lausanne, Switzerland; 4 Clinical Department and Laboratory of Intensive Care Medicine, Department of Cellular and Molecular Medicine, Katholieke Universiteit Leuven, Leuven, Belgium; 5 Department of Medicine, University of Louisville School of Medicine, Louisville, KY USA; 6 Intensive Care Medicine, Hospital Universitario, 12 de Octubre, Instituto de Investigación imas12, Madrid, Spain; 7 Department of Intensive Care Medicine, The Queen Elizabeth Hospital, Woodville, SA Australia; 8 Department of Critical Care Research, Faculty of Medicine, Nursing and Health Sciences, Monash University, Melbourne, Australia; 9 Department of Intensive Care Medicine, Lucerne Cantonal Hospital, Lucerne, Switzerland; 10 Department of Anaesthesiology and Intensive Care, University of Tartu, Tartu, Estonia; 11 Ede and Division of Human Nutrition and Health, Gelderse Vallei Hospital, Wageningen University and Research, Wageningen, The Netherlands; 12 Division of Anaesthesiology and Intensive Care Medicine, Department of Clinical Science, Intervention and Technology, Karolinska Institutet, Stockholm, Sweden; 13 Department of Anesthesiology and Surgery, Duke University School of Medicine, Durham, NC USA

**Keywords:** Critically ill, Stress response, Energy metabolism, Muscle wasting, Sarcopenia, Refeeding syndrome, Gastrointestinal dysfunction

## Abstract

The preferential use of the oral/enteral route in critically ill patients over gut rest is uniformly recommended and applied. This article provides practical guidance on enteral nutrition in compliance with recent American and European guidelines. Low-dose enteral nutrition can be safely started within 48 h after admission, even during treatment with small or moderate doses of vasopressor agents. A percutaneous access should be used when enteral nutrition is anticipated for ≥ 4 weeks. Energy delivery should not be calculated to match energy expenditure before day 4–7, and the use of energy-dense formulas can be restricted to cases of inability to tolerate full-volume isocaloric enteral nutrition or to patients who require fluid restriction. Low-dose protein (max 0.8 g/kg/day) can be provided during the early phase of critical illness, while a protein target of > 1.2 g/kg/day could be considered during the rehabilitation phase. The occurrence of refeeding syndrome should be assessed by daily measurement of plasma phosphate, and a phosphate drop of 30% should be managed by reduction of enteral feeding rate and high-dose thiamine. Vomiting and increased gastric residual volume may indicate gastric intolerance, while sudden abdominal pain, distension, gastrointestinal paralysis, or rising abdominal pressure may indicate lower gastrointestinal intolerance.

## Introduction

The importance of nutrition in the critically ill is increasingly acknowledged, especially in patients with long stay in the intensive care unit (ICU), who often require prolonged life-sustaining support and go through a state of severe catabolism [1, 2]. Some aspects of the nutrition practice such as the preferential use of the early oral/enteral nutrition (EN) over «gut rest» and the acceptance of delaying provision of amounts of nutrients calculated to match the losses and expenditure, while other aspects can raise controversial views [3–5].

International guidelines have been recently updated by the American Society of Parenteral and Enteral Nutrition/Society of Critical Care Medicine [6] and the European Society of Clinical Nutrition and Metabolism (ESPEN) [2, 7], with various levels of supporting evidence (Table 1). A group of experts in critical care nutrition from different regions of the world was commissioned to discuss some of the practicalities of early EN, listed in Table 1 and supported in the corresponding sections, to use and to complement the guidelines [6, 7] by providing tips inspired by the current knowledge and clinical experience of the experts. Importantly, nutritional requirements will vary according to the phase of critical illness, our tips are general in nature, and an individualized approach should always be used.

**Table 1:** Guide to EN—summary table

	Question	Suggested answer	ASPEN/SCCM guidelines [6]	ESPEN guidelines [7]
1	When to start?	Start within 24–48 h of ICU admission	Recommendation: start early EN within 24–48 h (quality of evidence: very low)	Start early EN (within 48 h) rather than delaying EN (grade of recommendation: B strong consensus) Start early EN (within 48 h) rather than early PN (grade of recommendation: a strong consensus)
2	What to do in case of vasopressor agents?	Start low-dose enteral nutrition Hold EN for patients who are being actively resuscitated or unstable	Suggestion: in the setting of hemodynamic instability, hold EN until the patient is fully resuscitated and/or stable Consider initiation/reinitiation of EN with caution in patients undergoing withdrawal of vasopressor support (expert consensus)	EN should be delayed if shock is uncontrolled. Low-dose EN can be started as soon as shock is controlled, while remaining vigilant for signs of bowel ischemia [grade of recommendation: Good practice point (GPP)]
3	How to achieve enteral access?	Short-term (expected duration < 4 weeks): use nasogastric tube or postpyloric in case of delayed gastric emptying) Long-term (> 4 weeks): place percutaneous enteral access (gastrostomy or jejunostomy)	Suggestion: in most critically ill patients initiate EN in the stomach {Expert consensus} Recommendation: Infuse EN lower in the GI tract in patients who are at high risk for aspiration or with intolerance to gastric EN (quality of evidence: moderate to high)	Use gastric access as the standard approach to initiate EN (grade of recommendation: GPP strong consensus) Use postpyloric feeding in patients with gastric feeding intolerance not solved with prokinetic agents (grade of recommendation: B strong consensus) Consider postpyloric, mainly jejunal feeding in patients at high risk for aspiration (grade of recommendation: GPP strong consensus)
4	How much energy?	Accept below energy expenditure during the early phase and increase energy to match energy expenditure later (4–7 days)	Suggestion: patients at low nutrition risk with normal baseline nutrition status and low disease severity (e.g., NRS 2002 ≤ 3 or NUTRIC score ≤ 5) do not require specialized nutrition therapy over the first week of hospitalization in the ICU (expert consensus) Recommendation: Start either trophic or full nutrition by EN for patients with acute respiratory distress syndrome (ARDS)/acute lung injury (ALI) and those expected to have a duration of mechanical ventilation ≥ 72 h (quality of evidence: high) Suggestion: advance EN toward goal over 24–48 h while monitoring for refeeding syndrome in patients who are at high nutrition risk (e.g., NRS 2002 ≥ 5 or NUTRIC score ≥ 5, without interleukin 6) or severely malnourished (expert consensus)	Administer hypocaloric EN (not exceeding 70% of EE) in the early phase of acute illness (grade of recommendation: B strong consensus) Increase caloric delivery can be increased up to 80–100% of measured EE after day 3 (grade of recommendation: 0 strong consensus)
5	When should energy-dense formulas be used?	Use energy-dense formulas in patients with GI intolerance of full-volume isocaloric enteral nutrition, patients needing fluid restriction or during transitioning to oral nutrition (intermittent-feeding schedule)	No specific recommendation	No specific recommendation
6	How much proteins?	Low dose (e.g., 0.8 g/kg/day) during the early phase—to be increased to > 1.2 g/kg/day later	Suggestion: Administer sufficient (high-dose) protein in the range of 1.2–2.0 g/kg actual body weight per day and may likely be even higher in burn or multitrauma patients (quality of evidence: very low)	During critical illness, 1.3 g/kg protein equivalents per day can be delivered progressively (grade of recommendation: 0: strong consensus)
7	When should hyperprotein formulas be considered?	During the late stable phase—monitoring of renal function/acid–base status		
8	How and when to start micronutrient supplementation?	Thiamin upon admission—others when insufficient amounts by enteral nutrition	We suggest that a combination of antioxidant vitamins [including vitamins E and C (ascorbic acid)] and trace minerals (including selenium, zinc, and copper) in doses reported to be safe in critically ill patients be provided to those patients who require specialized nutrition therapy (quality of evidence: low)	No specific recommendation
9	How to screen and manage patients for refeeding syndrome?	Plasma phosphate levels at least once a day when starting enteral nutrition Low-dose enteral nutrition, supplemental thiamin and phosphate	Monitor closely serum phosphate concentrations and replace phosphate appropriately when needed suggestion: (expert consensus)	Electrolytes (potassium, magnesium, phosphate) should be measured at least once daily for the first week [grade recommendation: GPP strong consensus (92% agreement)] In patients with refeeding hypophosphatemia (< 0.65 mmol/ l or a drop of > 0.16 mmol/l), electrolytes should be measured 23 times a day and supplemented if needed [grade recommendation: GPP strong consensus (100% agreement)] In patients with refeeding hypophosphatemia energy supply should be restricted for 48 h and then gradually increased [grade recommendation: B strong consensus (100% agreement)]
10	How to assess gastrointestinal tolerance?	At the start of low-dose EN: high gastric residual volume (optional—threshold 500 ml/6 h), vomiting, pain, distension, elevated/increasing intra-abdominal pressure, absent bowel sounds—dynamic ileus	Suggestion: Do not use GRVs as part of routine care to monitor ICU patients receiving EN Suggestion: for those ICUs where GRVs are still utilized, avoid holding EN for GRVs < 500 mL in the absence of other signs of intolerance (quality of evidence: low)	No specific recommendation statement

## Question 1: When to start?

Critically illness induces a cascade of metabolic and hormonal derangements, leading to severe macro- and micronutrient deficiencies [8]. The provision of exogenous nutrients via early commencement of EN helps mitigate this catabolic state and prevent intestinal villi atrophy, enterocyte apoptosis, inflammatory infiltration, dysbiosis and impairment of gut immune functions [9]. Early EN may alleviate or even reverse some of these pathophysiologic cascades [10–12]. Clinical data have also supported early EN (within 24–48 h of ICU admission) in critically ill patients. Multiple meta-analyses of randomized controlled trials showed that early EN compared to late EN was associated with reduced infectious morbidity in ICU patients [5, 6, 13]. However, several of the studies were of small sample sizes, and some were conducted more than 3 decades ago, with different standards of ICU management and nutritional therapy. Additionally, some of the older trials have important methodological limitations questioning their internal validity including selection bias, frequent postrandomization exclusions and lack of adherence to the intention-to-treat principle. A recent Cochrane meta-analysis assessed current evidence to be of very low quality, leading to uncertainty as to whether early EN, compared with delayed EN, affects the risk of mortality, feed intolerance or gastrointestinal complications, or pneumonia [14].

In spite of the low level of evidence, recent guidelines [6, 7] recommend the commencement of low-dose EN within 24–48 h of critical illness in the majority of ICU patients.

## Question 2: What about EN in patients receiving vasopressor agents?

Patients receiving vasopressor agents represent a special group, in which the potential benefit of early EN should be balanced against the associated risk [15–17]. Some data suggest that trickle feeding is possible even with high dose norepinephrine [18]. Data regarding the clinical benefits and risks of early EN in patients on vasopressor agents are limited. Although several observational data described an association between early EN and bowel ischemia, establishing causality between vasopressor agents and bowel ischemia in these studies is difficult [19–21]. In the NUTRIREA-2 trial, adults receiving mechanical ventilation and vasopressor agents were randomized to either early parenteral nutrition (PN) or EN, both at rates calculated to match the energy expenditure [22]. The study found no difference in the primary endpoint of 28-day mortality, but demonstrated a fourfold increase in bowel ischemia and acute colonic pseudo-obstruction with early EN [22]. The study suggests that full dose EN should be postponed until hemodynamic stability is restored. Whether lower amounts of EN or no EN at all would be the best option in patients with severe shock is not known. Still, aggregated data from 11 randomized controlled trials (RCTs, *n* = 597) show that early EN might decrease infectious complications [14] presumably via the protection of gastrointestinal wall integrity [23]. Nevertheless, in NUTRIREA-2 (*N* = 2410) and CALORIES (*N* = 2400), early EN as compared to PN for no more than 3–5 days did not decrease the infection rate [22, 24].

Several related questions are currently subject to ongoing research including comparing early trophic EN with no EN in the first 24 h [25–29].

Given the existing data, low-dose EN is recommended within 48 h of ICU admission, in patients with controlled shock requiring small or moderate doses of vasopressor and delaying EN in patients who are actively being resuscitated or are unstable [5, 6]. In patients requiring vasopressors, EN should be started with gradual advancement, with monitoring for symptoms and signs of gastrointestinal intolerance or unexplained worsening hemodynamic status.

## Question 3: How to achieve enteral access?

Decisions regarding enteral access are often determined by local expertise, anticipated duration of feeding, and evidence of gastroparesis or impaired gastrointestinal transit [30].

Short-term feeding may be facilitated by blind bedside placement of a nasogastric tube. Reliable aids to confirm location within the stomach include an abdominal radiograph, continuous CO2 monitor, or differential esophageal/tracheal compliance to intermittent suction [30].

The decision to switch from gastric to postpyloric feeding is based on perceived intolerance or delayed gastric emptying [7]. The placement of a postpyloric tube can be achieved endoscopic techniques, blind corkscrew technique, or GPS-guided or optically-guided tubes [30]. Use of a magnet-directed or flanged Tiger tube should be avoided. Surgical or radiologic techniques require transport to the operating room or radiologic suite, respectively. Randomized trials show a switch to postpyloric feeding reduces pneumonia significantly, but arguably no other outcome benefits are incurred, thereby underlying controversial views [6, 31, 32]. The decision to switch from nasoenteric to percutaneous access is predicated on an anticipated duration of feeding of greater than four weeks. A size of percutaneous endoscopic gastrostomy (PEG) tube larger than 18–20 French should be avoided, as torsion on the side wall leading to enlarged stomal diameter is more likely. Surgical placement of a gastrostomy tube is preferred in the presence of ascites, excluded stomach following bariatric surgery, or altered postoperative anatomy. Radiologic placement of a gastrostomy tube utilizes a variant of the “Introducer” technique.

The decision to attain deep jejunal access, such as converting a PEG to a PEGJ, is based on evidence of delayed gastric emptying [30, 33]. A new PEG can be converted even at initial placement by shortening the length of the PEG and placing a second smaller jejunostomy tube (J-tube) through the PEG into the small bowel. A mature tract (> 7–10 days since initial placement) is required to place a one-piece PEGJ, which affords a larger lumen for both feeding and aspiration.

## Question 4: How much energy?

The concept of high enteral energy intake has been postulated and tested, but this strategy failed to improve vital -and long-term functional [34–36] outcome in large-scale RCT’s [37–40]. These results suggest that disease-related anorexia contributes less to preventing lean-tissue wasting early in critical illness, than inflammation and mobilization. In some ICU patients, early up to target EN may moreover provoke harm. In patients recovering from circulatory shock, it provoked a small but significant increase in potentially lethal ischemic bowel complications [22]. Following a transitory decrease in serum phosphate, a feeding strategy aiming to achieve nutritional target increased mortality as compared to early nutrient restriction [41] (Question 9). Based on lack of benefit in large heterogeneous populations and signals of harm in some studies, ESPEN guidelines advise against early rapid advancement of feeding to target EN [7].

The individualization of intakes rather than aiming for enhanced or more restrictive feeding, equally applied to all patients might physiologically make more sense. Several scores—integrating clinical characteristics upon ICU admission and/or biomarkers—have been constructed in order to identify patients who might benefit from earlier or enhanced nutrition support. In particular, the value of the NUTRIC (The Nutrition Risk in the Critically Ill) score appeared promising for that purpose in observational analyses [42], but was refuted in stratified sub-analysis of the PERMIT-RCT [43]. Indirect calorimetry (IC) provides accurate estimation of energy burned by patients at rest [7]. IC-guided nutrition therapy, however, did not convincingly improve outcomes, in the absence of methodologically sound evidence [44–47]. The individualization of feeding based on a biomarker is not yet validated [48, 49].

While the impact of early nutrition interventions in ICU appears to be limited, nutrient provision during in-ICU rehabilitation and after ICU discharge is unexplored and potentially relevant to functional recovery [1, 50, 51].

In summary, energy intakes should be lower than energy expenditure during the early phase (4–7 days) and are increased to match energy expenditure later.

## Question 5: When should energy-dense formulas be used?

The macro- and micronutrient content of EN differs between various formulations. While isocaloric EN (1 kcal/ml) is commonly prescribed to achieve estimated or measured caloric goals [52–54], energy-dense formulae (> 1 kcal/ml) are also available. The increase in energy is achieved with increases in the proportion of mainly factor, carbohydrate.

The most common reasons for prescribing an energy-dense formulation are either to increase calorie delivery in patients with gastrointestinal dysfunction, an inability to tolerate full-volume isocaloric EN, fluid restriction or transitioning to oral nutrition using an intermittent-feeding schedule (e.g., overnight) while ensuring adequate energy intake. However, several caveats exist.

First, higher osmolality and fat content in energy-dense formulations may further impair delayed gastric emptying via neurohumoral feedback mechanisms (e.g., cholecystokinin, glucagon-like peptide-1) and via duodenal osmoreceptors which decrease gastric emptying until the gastric and duodenal contents are iso-osmotic. These solutions might cause diarrhea via the stimulation of fluid secretion within the small intestine [55]. Finally, the delivery of energy-dense nutrition at a lower rate may have the unintended consequence of decreased water and protein administration.

Second, the early administration of an energy-dense formulation has not been shown to improve outcomes. A large study reported that a near 50% increase in calorie delivery with an energy-dense EN formulation did not improve mortality at any time point, organ support or 6-month quality of life and functional outcomes compared to a 1 kcal/ml formulation [36, 40]. Subgroup analysis also did not demonstrate any differences between the energy-dense and isocaloric EN groups. Energy-dense EN was associated with increased gastrointestinal intolerance and higher blood glucose levels.

## Question 6: How much protein?

**Fig. 1:**
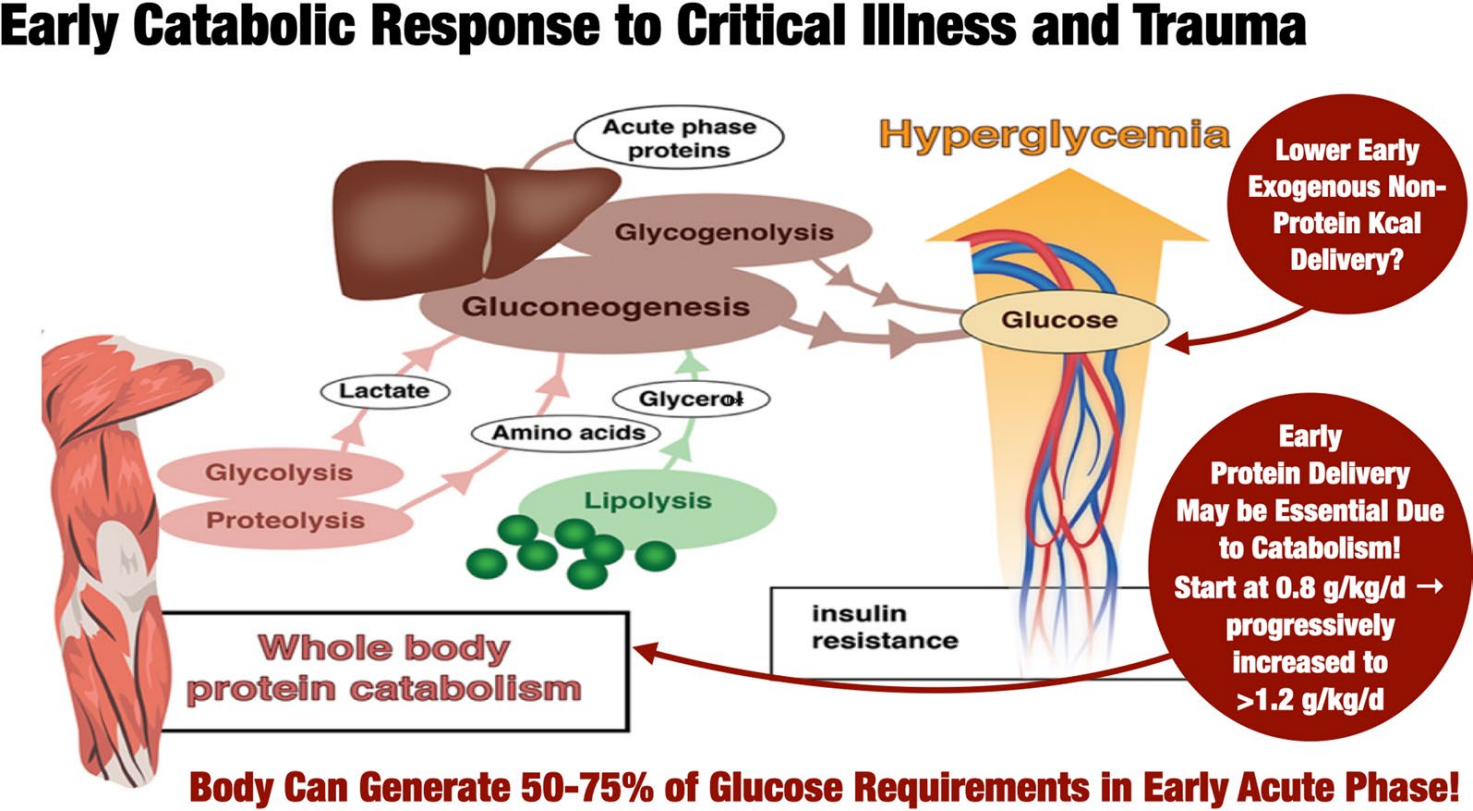
Acute phase catabolic response to critical illness and need for protein and non-protein calories. Adapted from: Ref. [57]

Patients’ muscle mass at ICU admission is correlated with ICU survival and this serves as an endogenous metabolic or amino acid reserve [4, 8, 56, 57] (Fig. 1). The catabolic response leads to marked muscle mass loss of up to 1 kg per day over the first 10 days of ICU stay and is associated with ICU-Acquired Weakness [58]. Nitrogen losses increase fourfold within the first 24 h of ICU stay [59].

Current data consistently demonstrate that ICU patients receive low amounts of protein (average of 0.6 g/kg/day for the first two weeks) [24, 60–67]. Higher protein provision is associated with reduced mortality in adults in observational trials [61, 68–74], biochemical outcome parameters and morphometric outcomes in skeletal muscle [75–79], improved quality of life at 3-month post-ICU [69] or handgrip strength at hospital day 7 and muscle mass [73]. However, prospective studies show limited effects on clinical, patient-centered and functional outcomes or yield negative results [45, 67, 74–81]. Admittedly, a limited number of large RCTs examined clinical outcomes of specifically increasing protein administration.

**Fig. 2:**
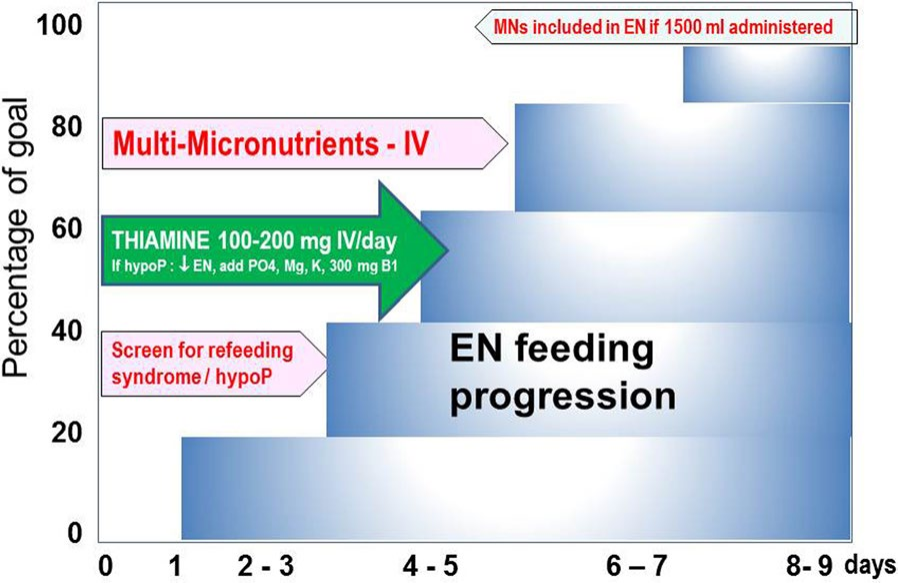
Recommendations for the progression of enteral nutrition delivery, micronutrients delivery and management of refeeding. Adapted from: CHUV Lausanne and Gelderse Vallei Hospitals. The *X* axis represents the time from admission (days, arbitrary example) and the *Y* axis the percentage of nutritional goal determined by a computer protocol using sex, height, weight (first 3 days) and later by indirect calorimetry or calculation prioritizing the avoidance of energy overfeeding. Regular (hourly) checks of intakes including the amount of non-nutritional energy (propofol, glucose, citrate) are recommended to adapt the infusion rate. Multi-micronutrients are administered IV until the dietary recommended intakes are met by the EN solution. The screening for refeeding syndrome is based on daily phosphate determination from day 2. In case of hypophosphatemia (hypoP) (serum phosphate (PO4) < 0.65 mmol/l, or a drop from baseline > 0.16 mmol/l occurring within 72 h of the start of EN) decrease the amount of energy delivered to a maximum of 500 kcal/day, supplement phosphate, magnesium (Mg) and potassium (K) and additional boluses of thiamine (vit B1, 500–1000 mg IV)

Hence, there is no evidence for a higher protein intake in critically ill patients in terms of clinically relevant outcomes in prospective randomized trials [82, 83]. Moreover, some harm can be related to excessive amounts of proteins in a post hoc analysis of prospective trials performed in adults [45, 84, 85] or in children [86] and in a retrospective study [87]. Hence, it may be prudent to start protein delivery at a lower dose (~ 0.8 g/kg) and ramp up protein dose to the targeted protein goal (> 1.2–1.3 g/kg/day [6, 7] (Fig. 2). However, this strategy was not previously evaluated in prospective studies.

The role of high-protein intakes that stress the need for focused larger clinical trial evidence examining the effect of specifically increasing protein delivery [4, 67, 84], combined with active mobilization to optimize physical therapy and functional outcomes in long-stayers, requires further study. Importantly, muscle volume and strength are not necessarily related. Preliminary data suggest that the combination of neuromuscular electrical stimulation and high-protein supplementation (1.8 g/kg/day) significantly improved short physical performance [88]. The role of high-dose protein delivery and in-bed ergometry (cycling) is being meaningfully studied [89] (NCT03021902).

## Question 7: When should hyperprotein formulas be used?

There now exist a range of available high protein-to-energy ratio products intended to meet protein targets and non-protein calorie goals with a limited risk of overfeeding of non-protein calories. The use of enteral protein supplements or supplemental amino acid solutions (such as clear liquid whey protein formulas) is proposed for this purpose [90]. However, it is important to keep the amino acid composition well balanced. Nutrition regimens that are grossly unbalanced inflict a metabolic strain on the patient [91]. A high-protein product may be used in the later stable phase of critical illness [85]. However, there are no data from prospective randomized controlled studies with clinically relevant outcomes to support this recommendation.

Some potential alternatives include the addition of the leucine metabolite HMB (hydroxy methyl butyrate) to improve amino acid metabolism and reduce net protein breakdown [92].

A high-nitrogen intake should always be accompanied by daily monitoring of plasma concentration of urea and creatinine together with base excess. If plasma urea concentration is increasing, urea excretion in urine should be identified and followed by a decrease of protein intake and eventually renal replacement therapy. If base excess increases, always consider reducing protein intake. Acidosis may come in critically ill patients for several reasons, but when the renal compensatory mechanisms are overridden will the ability to eliminate a surplus of nitrogen be impaired.

## Question 8: When and how to start micronutrients?

Ingestion of micronutrients (MN), i.e., trace elements and vitamins, is essential for normal metabolism [93], immunity [94], and antioxidant defense. They work as a web, and 24 of them are "essential," meaning that nutrition is the only source. The body stores of MNs are variable but generally insufficient to ensure normal metabolism beyond one week. The MNs needs will depend on the presence of prior deficiency, food intake before admission, particular body fluid losses, disease, and feeding rate. The available feeding products are meant to cover the needs of healthy people (dietary reference intakes) provided about 1500 kcal/day is delivered to the patients [95]. However, these amounts are not integrating the specific requirements of critically ill patients. Intestinal function and absorption are often absent or depressed during the first days, and antioxidant stress is maximal [96].

Further, most recent guidelines [7] recommend that EN is started within 48 h of admission after stabilization [5] and progressed to target over 3–4 days (Fig. 2). Consequently, MN delivery starts at close to zero and remains below DRI for nearly a week, or "forever" in patients receiving less than 1500 kcal. It has been proposed to measure blood concentrations of some MN at risk [15]. The results of analysis are often not timely available and may be costly. As most patients stay briefly (< 5 days), there is no time to adapt to a delayed abnormal result. Nevertheless, blood values determination is rational for selected MNs depending on pathology and treatment when the patients stay more than a week, especially when renal replacement therapy is required [97–101].

Critically ill patients are often admitted with a nutritional deficit developed in the days preceding ICU admission, translating into MN deficiencies. The earliest manifestation is refeeding syndrome (RFS), with thiamine being in the first line discussed below [105]. The late complications are less specific, generally unrecognized, and sometimes called an "invisible foe" [110, 102–105]. Infections and wound healing complications are in the first line as MN are essential for immune defense. Therefore, during the early phase, as EN cannot cover the everyday needs and the higher needs associated with critical illness, early intravenous delivery of doses like those used in PN is rational (1 vial multitrace element and multivitamin + 100–200 mg thiamine) (Fig. 2). A few trials have shown that the strategy to deliver MNs intravenously at doses 4–5 times higher than for PN until EN can cover the needs is associated with better global outcomes [106, 107].

## Question 9: How to screen and manage patients for refeeding syndrome?

Refeeding syndrome (RFS) is a potentially fatal acute metabolic response following the reintroduction of nutrients after a variable length of starvation that may lead to morbidity and increased mortality [108].

Refeeding syndrome is characterized by electrolyte shifts that arise from a switch from a catabolic state using fat and protein as energy sources back to carbohydrate metabolism. Glucose substrate utilization leads to increased insulin levels, resulting in thiamine depletion and low plasma levels of phosphate, magnesium and potassium due to the intracellular shift of electrolytes [109–111]. The complications of RFS are so severe that the liberal administration of intravenous thiamine 100–200 mg/day for the first 3 days should be part of routine (Fig. 2). In the absence of appropriate management, many clinical potentially life-threatening consequences may develop [108].

Due to significant variations in RFS definitions, its exact incidence remains unknown. However, when RFS is defined by hypophosphatemia (hypoP) with a cut-off level of 0.65 mmol/L, the incidence ranges from 34 to 40%, with 4–10% presenting severe hypophosphatemia (phosphate < 0.32 mmol/L) or a drop after the start of glucose infusion or nutrition therapy [111–113]. Most recent studies in ICU patients using hypoP as the primary criterion to define RFS did not identify clinical predictors of RFS on ICU admission [41, 110, 114]. Therefore, all critically ill patients should be considered at risk of refeeding syndrome and monitored for serum phosphate levels at least once a day when starting EN [110]. The diagnostic criteria and recommendations to monitor phosphate have recently been adopted by the ESPEN nutrition guidelines [7].

Recent studies have demonstrated that high-energy intake during RFS is associated with increased mortality, and caloric restriction confers improved outcomes [114, 115]. The difference in mortality occurred much later during patients’ ICU stay after correction of electrolyte imbalance, suggesting a complex pathophysiology [41, 114]. Thiamine administration and caloric restriction of 500 kcal/day or 25% of the estimated target inspired from NICE guidance [116] is a frequent practice for ICU patients with hypoP/RFS for at least 48 h.

Practical protocols are available on-line (e.g., [117]) to guide progressing energy to target in the early phase of ICU stay is provided. Energy target on admission is based on predictive equations. In 4 steps of 25%, feeds are advanced to the estimated target to prevent overfeeding, including non-nutritional energy from propofol and citrate. Indirect calorimetry is performed to adjust to the actual energy expenditure and set as a new target. When refeeding hypoP within 72 h after the start of EN is encountered, caloric restriction is warranted. After 48 h subsequently, the following steps (25%) are set.

## Question 10: How to assess gastrointestinal intolerance?

**Fig. 3:**
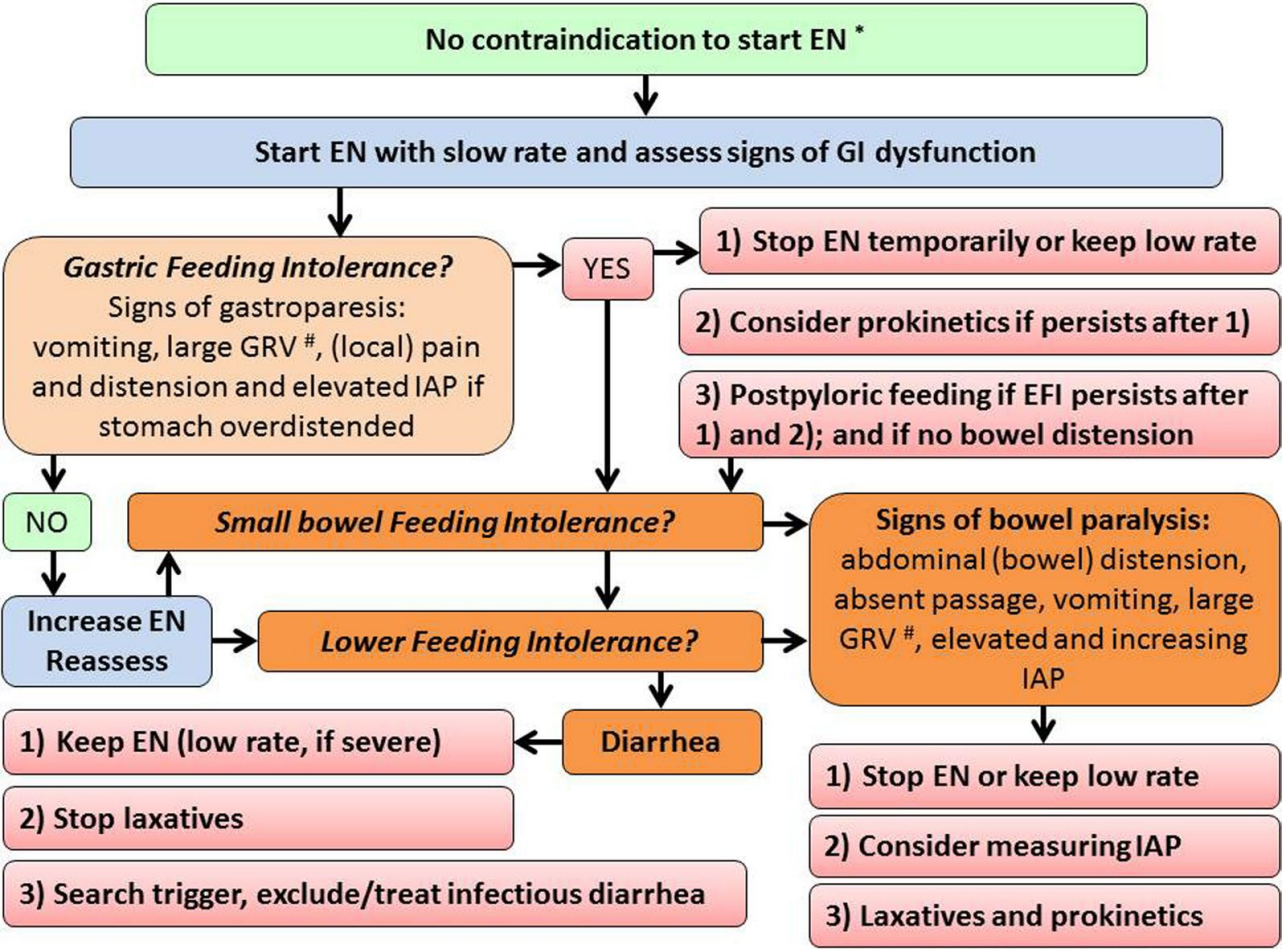
Screening for enteral feeding intolerance (ARB). Differentiation between EFI in different parts of GI tract and respective terminology has not been uniformly established. *Suggested contraindications to EN are uncontrolled shock, uncontrolled hypoxemia and acidosis, uncontrolled upper GI bleeding, gastric aspirate > 500 ml/6 h, bowel ischemia, bowel obstruction, abdominal compartment syndrome, and high-output fistula without distal feeding access. # GRV between 200 and 500 ml can be considered increased and > 500 ml a cut-off for discontinuation of EN

Gastrointestinal (in)tolerance is often defined with certain symptoms/signs, with ‘tolerance’ meaning the absence of these symptoms and signs [118–120]. ‘Enteral feeding intolerance’ (EFI) is commonly defined as a certain amount of gastric residual volumes (GRV) [119–121], capturing only upper gastrointestinal (GI) problems after initiation of enteral tube feeding, while both upper and lower parts of the GI tract can be involved (Fig. 3). In most of available studies, patients with EFI were more severely ill compared to patients tolerating EN, suggesting that EFI could be an epiphenomenon or a marker of disease severity [118]. In several studies, the occurrence of EFI as a feature of GI dysfunction was shown to independently associate with adverse outcome, as an additional organ dysfunction [119, 121–124].

Gastric intolerance assessed by the GRV measurement is the prevalent gastrointestinal symptom in ICU patients treated with EN [118, 125, 126]. Measurements of GRV have been omitted in many sites since a study showed no benefit of GRV-guided EN in patients with already established EN despite vomiting occurred more often in patients without than with GRV measurements [127]. However, the relation of GRV with the tracheal aspiration of gastric contents and pneumonia development is not clear [128, 129] and GRV measurement is a time-consuming practice and is associated with infectious risk (COVID-19) and variability in practices [121, 122]. Due to these factors and uncertainties, recent guidelines either do not recommend routine measurement of GRV [6], or suggest restricting GRV measurements to the initiation and progression of EN only [5, 7]. The latter is important, as evidence from RCTs is available only for medical patients having full EN already established at study inclusion [126]. Moreover, there is no good substitute for GRV, which could be considered as a surrogate marker of gastric emptying at bedside [128]. Therefore, depending on local constraints, GRV can still be included in assessment of EFI and a GRV over 500 ml/6 h is considered as an indication for intervention (delay or interruption of EN or application of prokinetics) [129]. [5, 7, 130–133], even though prokinetics has not been proven to improve patient-relevant outcomes [134].

Lower parts of GI tract are often involved, even in the absence of upper GI intolerance. Lower GI intolerance requires different management. Bowel paralysis leading to bowel distension in patient receiving EN may be associated with adverse outcomes. Patients in shock receiving early full EN compared to PN more often developed Ogilvie’s syndrome and bowel ischemia [22]. Monitoring and management of EFI and GI dysfunction is complicated due to the lack of robust and reproducible markers and multifaceted clinical presentation [49]. As no single straightforward marker reliably detects GI dysfunction, using composite scores combining several symptoms and signs could be helpful and should be considered [131]. EFI at the bedside is defined as features of GI dysfunction appearing during EN and consequently leading to reduction or discontinuation of EN. [123, 124, 135] Evidence on management options, unanswered issues and proposals for future research on GI dysfunction have been recently summarized [136]. In brief, patients should be carefully assessed for high gastric residual volume (optional—threshold 500 ml/6 h), vomiting, pain, distension, elevated/increasing intra-abdominal pressure, GI paralysis.

## Conclusions

The importance of medical nutrition in the care of the critically ill cannot be overstated. Overall, the management of EN requires a systematic and updated approach involving all ICU professionals, including practical approaches proposed in this document and regular updates. Auditing changes in practice are needed locally from the entire community of ICU professionals to increase the safety and efficiency of the delivery of EN.

## Data Availability

Not applicable.
